# Survival analysis of implants after surgical treatment of peri-implantitis based on bone loss severity and surgical technique: a retrospective study

**DOI:** 10.1186/s12903-023-02981-5

**Published:** 2023-05-22

**Authors:** Sooshin Hwang, Hee-min Lee, Pil-Young Yun, Young-Kyun Kim

**Affiliations:** 1 Department of Oral and Maxillofacial Surgery, Section of Dentistry, Seoul National University Bundang Hospital, 82 Gumi‑ro 173beon‑gil, Bundang‑gu, 13620 Seongnam Korea; 2 Department of Dentistry and Dental Research Institute, School of Dentistry, Seoul National University, 101, Daehak-ro Jongno-gu, Seoul, 03080 Korea

**Keywords:** Peri-implantitis, Surgical treatment, Bone loss rate, Surgical method, Survival analysis

## Abstract

**Background:**

Few trials have compared the results of surgical treatment for peri-implantitis based on severity of peri-implantitis and surgical method. This study investigated the survival rate of implants based on type of surgical method used and initial severity of peri-implantitis. Classification of severity was determined based on bone loss rate relative to fixture length.

**Methods:**

Medical records of patients who underwent peri-implantitis surgery from July 2003 to April 2021 were identified. Classification of peri-implantitis was divided into 3 groups (stage 1: bone loss < 25% (of fixture length), stage 2: 25% < bone loss < 50%, stage 3: bone loss > 50%) and performance of resective or regenerative surgery was investigated. Kaplan-Meier survival curves and Cox hazards proportional models were used to analyze the cumulative survival rate of implants. Median survival time, predicted mean survival time, hazard ratio (HR), and 95% confidence interval (CI) were calculated.

**Results:**

Based on Kaplan-Meier analysis, 89 patients and 227 implants were included, and total median postoperative survival duration was 8.96 years. Cumulative survival rates for stage 1, 2, and 3 were 70.7%, 48.9%, and 21.3%, respectively. The mean survival time for implants in stage 1, 2, and 3 was 9.95 years, 7.96 years, and 5.67 years, respectively, with statistically significant difference (log-rank p-value < 0.001). HRs for stage 2 and stage 3 were 2.25 and 4.59, respectively, with stage 1 as reference. Significant difference was not found in survival time between resective and regenerative surgery groups in any peri-implantitis stage.

**Conclusions:**

The initial bone loss rate relative to the fixture length significantly correlated with the outcome after peri-implantitis surgery, demonstrating a notable difference in the long-term survival rate. Difference was not found between resective surgery and regenerative surgery in implant survival time. Bone loss rate could be utilized as a reliable diagnostic tool for evaluating prognosis after surgical treatment, regardless of surgical method used.

**Trial registration:**

Retrospectively registered. (KCT0008225)

## Background

The long-term effectiveness of implant therapy for up to 20 years has been proven, but the risk of biological complications represented by peri-implantitis remains a major concern [1]. According to the consensus from the 2017 World Workshop on Periodontal and Peri-implant Diseases and Conditions, peri-implantitis is defined as a plaque-associated pathological condition occurring in tissues around dental implants, characterized by inflammation in the peri-implant mucosa and subsequent progressive loss of supporting bone [2, 3]. However, despite the above consensus, specific diagnostic criteria have not been unified and differed in each study since the concept of peri-implantitis first appeared in the 1980s [4]. In particular, different diagnostic criteria associated with bone loss have been described in numerous papers. Differences in diagnostic criteria significantly affect the prevalence of peri-implantitis and evaluation of implant prognosis [5, 6]. Furthermore, attempts to determine the prognosis of treatment after classifying peri-implantitis based on severity are scarce. Some suggestions have been made regarding classification of peri-implantitis, but consensus has not been reached to date [7, 8].

Despite the limitations of non-unified diagnostic criteria, studies on the treatment of peri-implantitis have been conducted. Clinicians determined that peri-implantitis increases when left untreated and progresses more rapidly than periodontitis in natural teeth [3]. Non-surgical conservative treatment (e.g., mechanical debridement, systemic antimicrobials, lasers) tends to be the first treatment option; however, therapeutic effects are unpredictable [9]. In particular, surgical treatment (e.g., resective surgery, regenerative surgery, combined resective and reconstructive surgery) reportedly show a better outcome in cases of serious peri-implantitis with severe bone loss [10–13].

Accordingly, many studies on surgical treatment and prognosis for peri-implantitis have been conducted. However, most have focused on the improvement of clinical parameters such as probing depth (PD) and treatment success after surgery and not on long-term survival. Roccuzzo et al. reported 10-year follow-up results after regenerative surgical treatment; however, due to the limited sample size and the number of patients lost to follow-up, the results regarding implant survival were inconclusive [10, 14–18]. In addition, most of the studies in which prognosis after surgical treatment was investigated have only considered resective surgery [10, 19–21]. Prognosis comparison between resective and regenerative surgery with long-term follow-up is rare, and results showed unclear difference in prognosis [14].

Therefore, according to Stuart’s 2012 peri-implantitis classification [8], we retrospectively evaluated the prognosis of surgical treatment based on the ratio of bone loss to length of the implant fixture and compared the difference of treatment outcomes between resective and regenerative surgery.

## Methods

### Study subjects

The Institutional Review Board of Seoul National University Bundang Hospital approved this study (B-2207-768-102), and study is retrospectively registered(KCT0008225). The study included patients who visited the Oral and Maxillofacial Surgery Department of Seoul National University Bundang Hospital from July 2003 to April 2021 with chief complaints of implant-related discomfort and who had undergone surgery for one or more implants diagnosed with peri-implantitis. The inclusion criteria for this study were (1) presence of clinical peri-implantitis symptoms (PD ≥ 4 mm in two or more aspects around the implant, bleeding and/or suppuration when probing, ≥ 2 mm of bone loss based on radiographic data) and history of surgical treatment (treatment accompanied with flap elevation; regenerative or resective surgery) and (2) presence of radiographic data before and after surgery and during the follow-up period. The exclusion criteria were (1) underwent only non-surgical treatments; (2) no follow-up data or radiographic data after surgical treatment; and (3) more than two surgical treatments with the same implant.

The following information was collected from the medical records and radiographic records: gender, age, systemic diseases, smoking habits, implant location, diameter and length of implant, type of implant, surgical method when placing the implant, bone graft during implant placement, types of bone graft material and membrane, time of implant placement, implant prosthesis loading time, type of prosthesis, time of diagnosis of peri-implantitis, peri-implantitis surgery method used, time of peri-implantitis surgical treatment, amount of implant bone loss at the time of surgical treatment, most recent follow-up time, and time of implant loss or removal (Table 2).

### Surgical treatment

Two types of surgical treatments were performed: resective and regenerative. If bone loss around the implant showed a tendency to progress horizontally, resective surgery was performed. If bone loss showed locally vertical progression or surrounding bone walls were all present, regenerative surgery was performed. The diagnosis, surgical treatment, and postoperative management were all performed by one experienced oral and maxillofacial surgeon. As a pre-surgical measure, conservative treatments such as peri-implant curettage, chlorhexidine irrigation, and antibiotic therapy (local and/or systemic) were performed. If surgical access was difficult due to the structure of the prosthesis, the surgery was performed after temporarily removing the prosthesis for easier access.

**Fig. 1:**
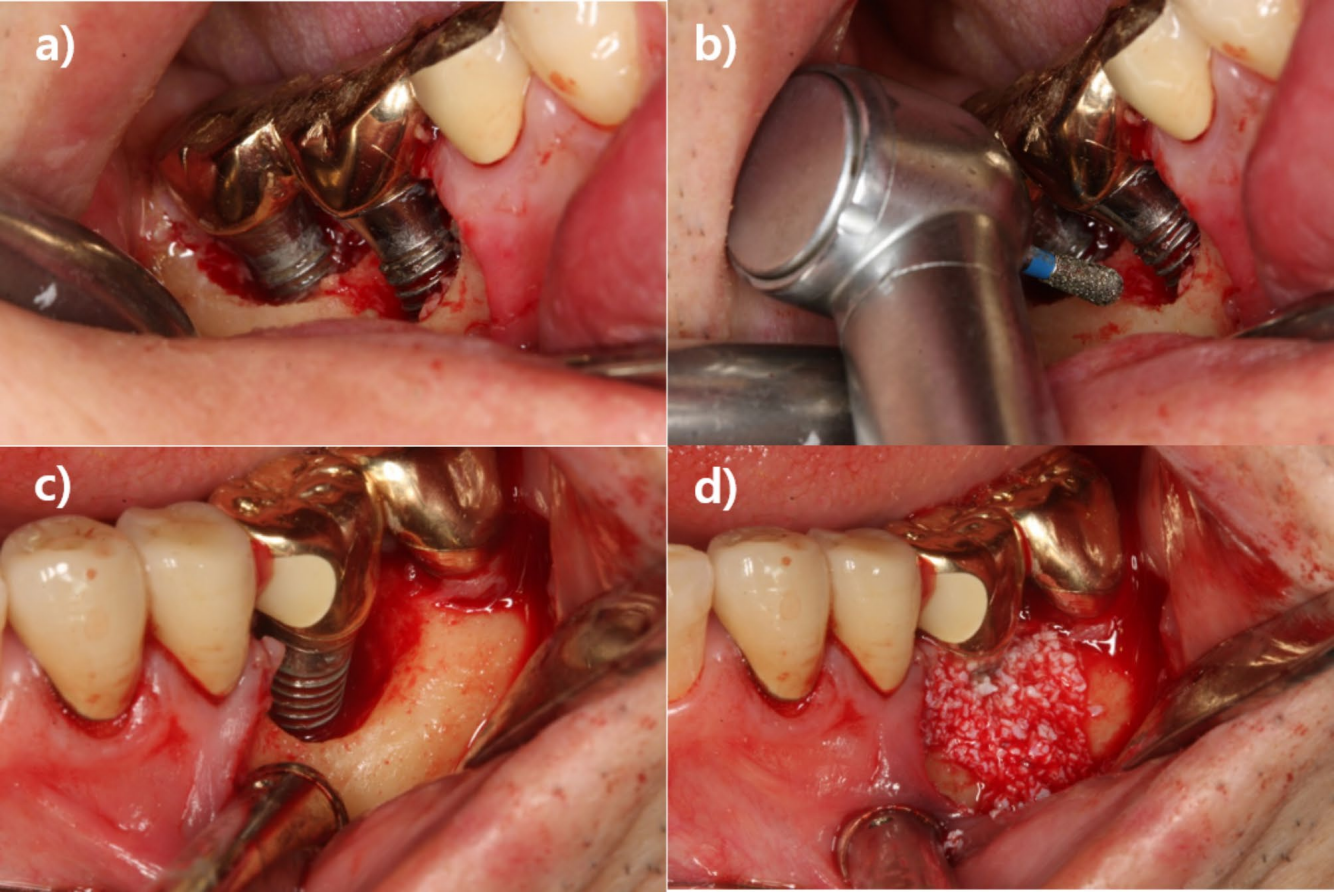
Clinical example of peri-implantitis surgical treatment; (a,b) resective surgery; (c,d) regenerative surgery

In resective surgery, an apically positioned flap (APF) was performed for pocket elimination. Full-thickness flap elevation was performed around the affected implant to sufficiently expose the implant surface and lesion. The surgeon performed implantoplasty selectively in the surgically accessible area that has lost osseointegration. In cases with an angular bony defect, instruments such as round bur, diamond bur, and bone file were used for osseous re-contouring. Inflammatory granulation tissue was removed using ultrasonic instruments, hand curette, and titanium brush (iBrush, Neobiotech, Seoul, Korea) cleansing, and the implant surface was cleansed and decontaminated using laser (Er-YAG laser therapy coding as peri-implantitis treatment: tip; 2061-cylinder, Energy;100 mJ, frequency; 10 Hz, Irrigation on, Manufactured by K.e.y Laser®, KaVo, Biberach, Germany), then applying tetracycline solution for 5 min, and cleaned with sterile saline solution. After injecting minocycline ointment around the fixture, the wound was closed, and a periodontal pack was attached. Regenerative surgery was performed using the same method for pocket elimination, including osseous re-contouring and mechanical cleansing and decontamination of the implant surface. Then, bone graft (autograft, allograft, or xenograft) was performed around the peri-implant bony defect before wound closure, with barrier membrane in some cases. APF was selectively performed when supra-alveolar defects without bone walls and locally vertical defect with bone walls both existed as combined form. Minocycline ointment was injected around the fixture, and the wound was closed with a periodontal pack, similarly to resective surgery. (Fig. 1)

### Peri-implantitis classification

Peri-implantitis was classified according to Stuart, 2012 [8]. Using the radiographic results obtained before the surgical treatment, implants were classified based on the ratio of amount of bone loss to fixture length. The three categories were as follows: bone loss < 25% of implant length (stage 1, Early), bone loss 25–50% of the implant length (stage 2, Moderate), bone loss > 50% of implant length (stage 3, Advanced; Table 1). For classification, intraoral periapical radiographs performed with the paralleling technique on a digital intraoral radiographic device (Heliodent Sirona, Sirona Dental Systems Inc., NY, USA) and measurement system (PACS, INFINIT Co., Seoul, Korea) were used. Bone loss ratio was determined using the mean distances from the mesial and distal aspects of the implant shoulder to the implant-bone contact points.

**Table 1:** Classification of peri-implantitis

*Stage 1* **(Early)**		Bone loss < 25% of the implant length† and PD ≥ 4 mm (bleeding on probing and/or suppuration*)		
*Stage 2* **(Moderate)**		Bone loss 25–50% of the implant length† and PD ≥ 4 mm (bleeding on probing and/or suppuration*)		
*Stage 3* ** (Advanced)**		Bone loss > 50% of the implant length† and PD ≥ 4 mm (bleeding on probing and/or suppuration*)		

†Noted on two or more aspects of the implant with bone loss ≥ 2 mm recorded.

*Measured on radiographs from time of definitive prosthesis loading to current radiograph. If not.

available, the earliest available radiograph following loading was used [8].

### Data analysis

IBM SPSS Statistics version 25.0 (SPSS Inc., Chicago, IL, USA) was used for statistical analysis with 95% statistical significance level. Time-to-event (i.e., time from surgical treatment to implant removal) was recorded by year after grouping based on peri-implantitis stage and surgical method used. Kaplan-Meier analysis was performed to obtain the survival curve, predicted mean survival time, and median survival time with 95% confidence interval (CI). Log-rank test was performed to compare results of each group if a significant difference was found using Bonferroni correction method. Then, Cox proportional hazards model regression analysis was performed to determine whether peri-implantitis stage is the factor affecting implant survival rate, and hazard ratio (HR) was calculated with 95% CI. To perform Cox proportional hazards regression analysis, the proportional hazards assumption (constant ratio of hazards between groups during the study period) needs to be valid. Thus, intersection of any Kaplan-Meier curve at any point was examined, and the observed *versus* expected plots method comparing the overall form of Cox proportional hazards regression model and Kaplan-Meier curve was used to determine the validity of the assumption [22–24].

## Results

**Table 2:** Demographic and examination data for patients diagnosed with peri-implantitis and treated with surgery

**Patients, n**	101
Mean age ± SD (range), years	68.2 ± 10.6 (40–92)
Gender, n (%)	
Male	56 (55.4)
Female	45 (44.6)
Smoking habits, n (%)	22 (21.8)
Systemic diseases, n (%)	72 (71.3)
Implants, n	**266**
Jaw, n (%)	
Maxilla	139 (52.2)
Mandible	127 (47.8)
Location, n (%)	
Anterior (incisor-1st premolar)	58 (21.8)
Posterior (2nd premolar-3rd molar)	208 (78.2)
Type and number of implants (%)	
Internal connection (Bone level)	108 (40.6)
Internal connection (Tissue level)	15 (5.6)
External connection	42 (15.8)
Other	17 (6.4)
Unknown*	84 (31.6)
Accompanied technique during implant placement, n (%)	
None	26 (9.8)
GBR (without sinus elevation)	113 (42.6)
Sinus elevation	47 (17.7)
Unknown*	80 (30.1)
Method of implant placement, n (%)	
Non-submerged (1 stage)	42 (15.8)
Submerged (2 stage)	138 (51.9)
Unknown*	86 (32.3)
Types of prosthesis, n (%)	
Single	23 (8.6)
Bridge	232 (87.2)
Full-mouth fixed hybrid prosthesis	8 (3.0)
Overdenture	3 (1.1)
Months from 1st surgery to prosthesis delivery date, mean ± SD	6.8 ± 3.5

* Data of the implants not placed in Seoul National University Bundang Hospital were unattainable and marked as unknown; SD, standard deviation

**Table 3:** Examination data after surgery based on peri-implantitis stage

Surgical group	Original group of patients and implants treated with peri-implantitis surgery	Patient file sample included in protocol analysis
Patients	101	89
Implants	266	227
Peri-implantitis stage before surgery		
1	88	79
Resective surgery (%)	52 (59.1)	47 (59.5)
Regenerative surgery (%)	36 (40.9)	32 (40.5)
2	89	70
Resective surgery (%)	46 (51.7)	35 (50)
Regenerative surgery (%)	43 (48.3)	35 (50)
3	89	78
Resective surgery (%)	26 (29.2)	20 (25.6)
Regenerative surgery (%)	63 (70.8)	58 (74.4)
Resective surgery, Total (%)	124 (46.6)	102 (44.9)
Regenerative surgery, Total (%)	142 (53.4)	125 (55.1)
Number of implants with peri-implantitis per patient, mean ± SD (range)		2.7 ± 1.8 (1–13)
Time (years) from implant placement to peri-implantitis diagnosis, mean ± SD (range)		4.0 ± 2.9 (0.2–11.5)
Function time (years) before peri-implantitis diagnosis, mean ± SD (range)		3.5 ± 2.9 (0–11.0)
Function time (years) before surgery, mean ± SD (range)		5.5 ± 3.3 (0.5–15.7)
Time (years) from peri-implantitis diagnosis to surgery, mean ± SD (range)		1.3 ± 1.7 (0–11.2)

* The patients who did not visit the clinic postoperatively and had no radiographic data after the surgery (13 implants) and implants surgically treated more than once (26 implants) were excluded from the original group; SD, standard deviation

From the original group of 266 implants in 101 patients, 227 implants in 89 patients were finally included in the study analysis after excluding implants in patients who did not visit the hospital postoperatively (n = 13) and implants surgically treated more than once (n = 26). Among the implants, 79 were stage 1, 70 stage 2, and 78 stage 3. Resective surgery was performed for 102 implants (44.9%) and regenerative surgery for 125 implants (55.1%; Table 3). The mean follow-up period after surgery was 4.3 years (± 3.4 years); 71 implants (31.3%) failed, and 156 implants (68.7%) were censored (survival or survival unknown).

**Fig. 2:**
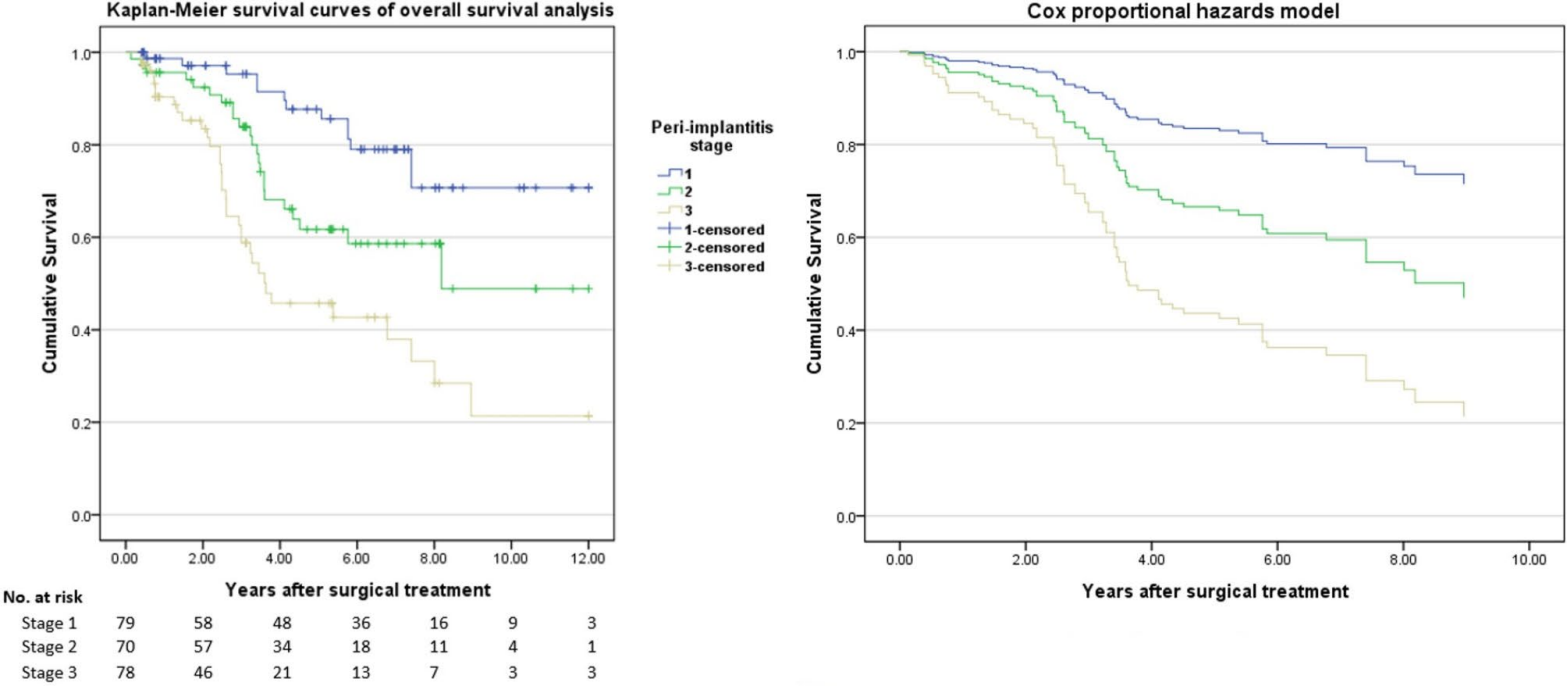
Kaplan-Meier(Log-rank test p ≤ 0.016) and Cox proportional hazards models(p ≤ 0.05) for implant survival analysis after surgical treatment

Table 4 shows the results of survival analysis based on peri-implantitis stage at the time of surgery obtained from Kaplan-Meier and Cox proportional hazards models. The median survival time of the implants was 8.96 years, and the final cumulative survival rate was 47.8% (36.8?58.8%). The 1-year survival rate was 94.9% (stage 1, 98.6%; stage 2, 95.6%; stage 3, 90.4%), the 3-year survival rate was 79.8% (stage 1, 95.3%; stage 2, 83.9%; stage 3, 58.8%), and the 5-year survival rate was 65.7% (stage 1, 87.7%; stage 2, 61.7%; stage 3, 45.7%). Stage 1 had a cumulative survival rate of 70.7% (55.8?85.6%) and mean predicted survival time of 9.95 years. Stage 2 had a cumulative survival rate of 48.9% (27.9?69.9%) and mean predicted survival time of 7.96 years. Stage 3 had a cumulative survival rate of 21.3% (4.2?38.4%) and mean predicted survival time of 5.67 years. A statistically significant difference was observed in survival time based on stage of peri-implantitis (log rank test, p-value < 0.001). In addition, after confirming the proportional hazards assumption was satisfied (Fig. 2), Cox hazard regression analysis was performed. Consequently, HR for stage 2 was 2.25 (p = 0.02) and for stage 3 was 4.59 (p < 0.001) when HR for stage 1 was set as a reference.

**Fig. 3:**
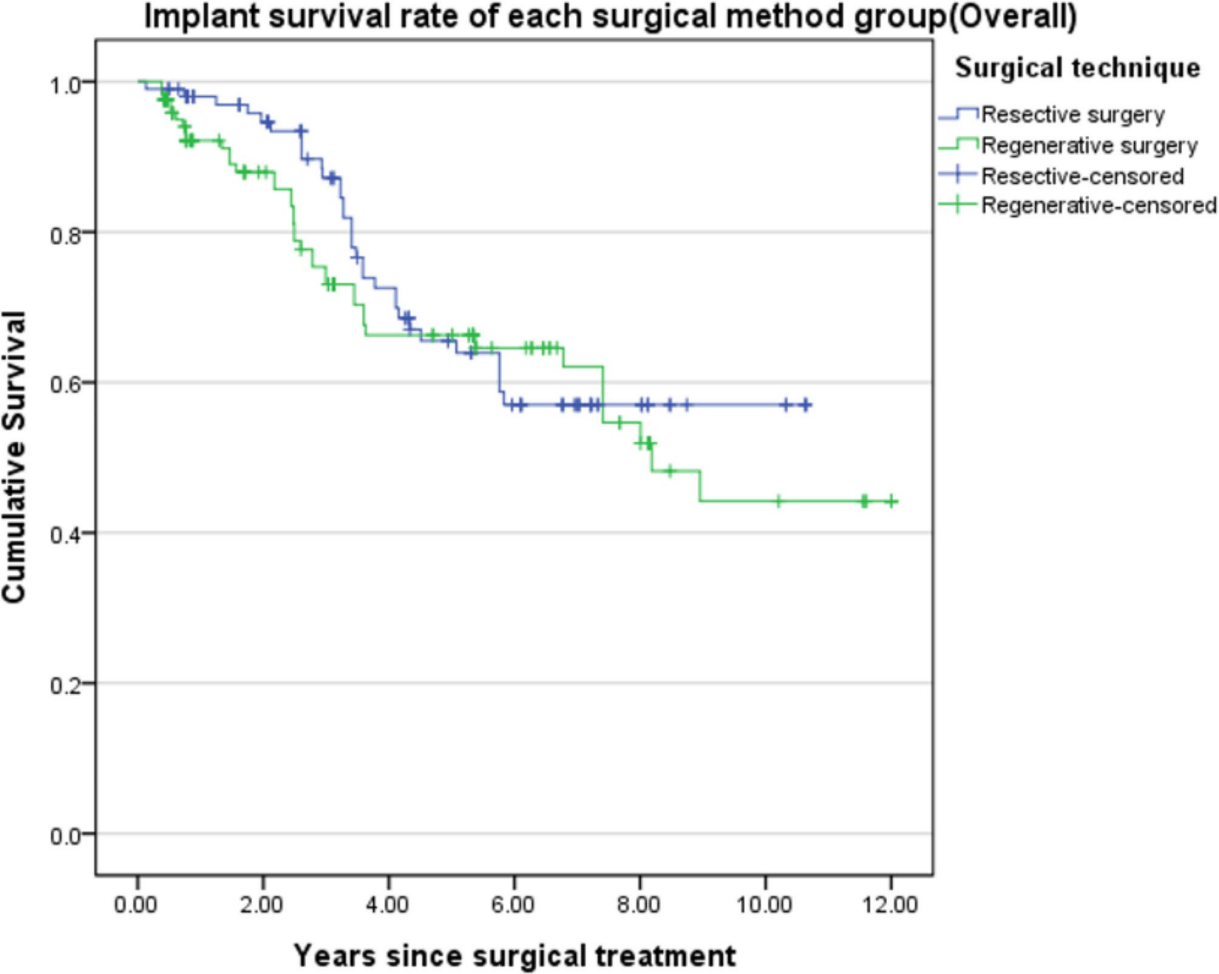
Kaplan-Meier curve for comparing the survival of implants by surgical method (Log-rank test) p = 0.444)

**Fig. 4:**
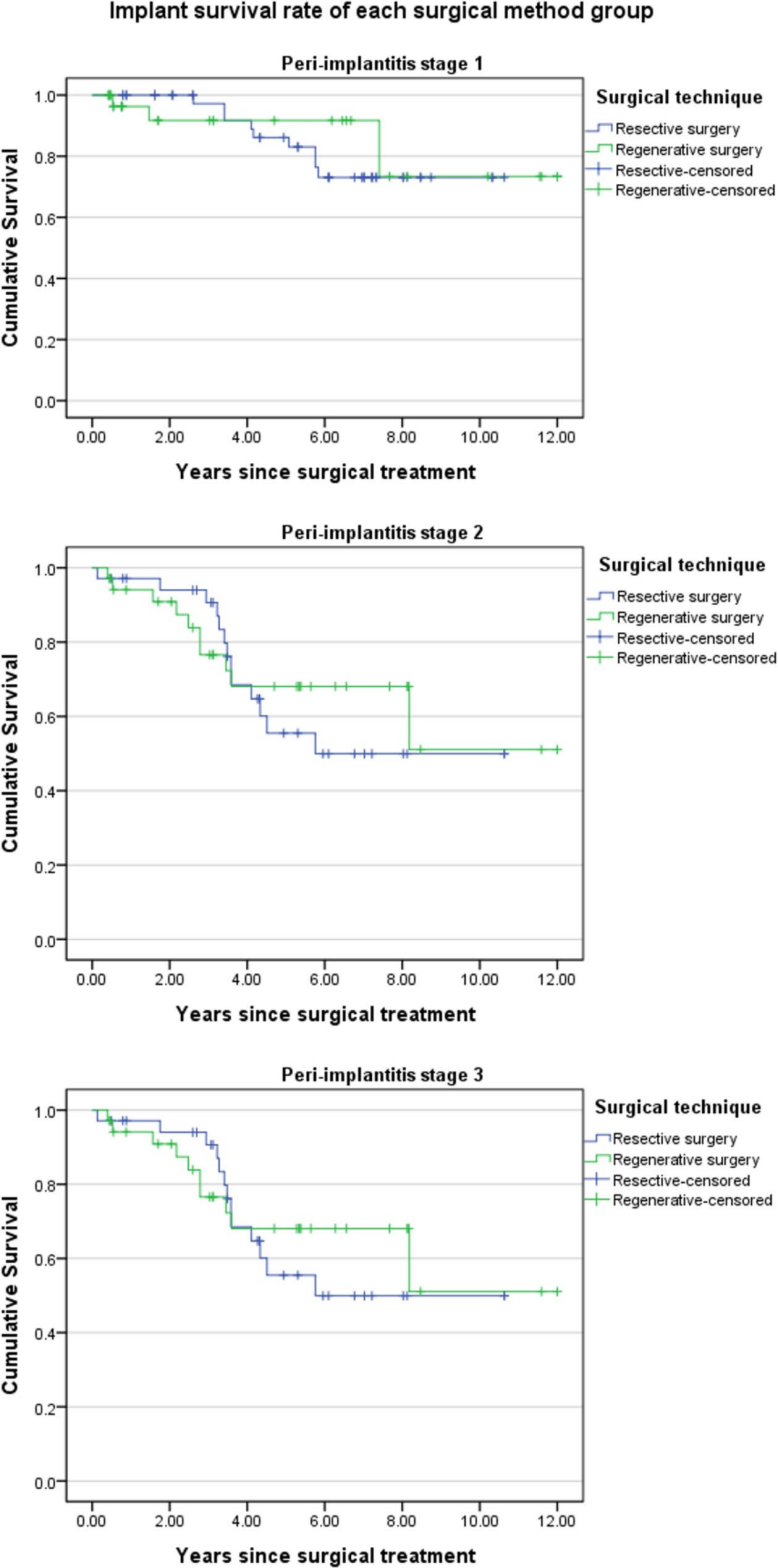
Kaplan-Meier curves of each peri-implantitis stage (Log-rank test p = 0.663 < Stage 1>, p = 0.675 < Stage 2>, p = 0.358 < Stage 3>

The cumulative survival rate in the group that underwent resective surgery was 57.0% (45.4–68.6%) compared to 44.2% (39.3–59.1%) for regenerative surgery; the difference was not significant (log rank test: p-value = 0.444). Significant difference was also not observed in any stage (stage 1 p-value = 0.663, stage 2 p = 0.675, stage 3 p = 0.358). The result was confirmed from Kaplan-Meier curves of the groups, which intersected at some points and not being clearly distinguished from each other. The result showed that surgical method did not affect the survival rate of implants after surgery, in any stage as well(Figs. 3 and 4).

**Table 4:** Survival estimate analysis (Kaplan-Meier and Cox hazard regression) based on peri-implantitis stage and surgical technique

Subgroup	Number	No. of Failures (%)	Cumulative survival rate (95% CI)	Median survival time after surgery in years	Estimated mean survival time after surgery in years (95% CI)	P-value (log-rank test)		HR (95% CI), p-value			
1	79	13 (16.5)	**70.7 (55.8–85.6)**	-*	9.95 (8.98–10.92)	< 0.001*	1.00 (Reference)				
Stage 1, Resective surgery	47	9 (19.1)	73.1 (57.8–88.4)		8.99 (8.06–9.93)	0.663					
Stage 1, Regenerative surgery	32	4 (12.5)	73.4 (48.9–97.9)		10.25 (8.73–11.77)						
2	70	23 (32.9)	**48.9 (27.9–69.9)**	**8.18**	7.96 (6.65–9.26)	< 0.001*	2.25 (1.14–4.45), 0.02*				
Stage 2, Resective surgery	35	13 (37.1)	49.9 (29.7–70.1)		7.11 (5.70–8.52)	0.675					
Stage 2, Regenerative surgery	35	10 (28.6)	51.1 (19.3–82.9)		8.26 (6.38–10.14)						
3	78	35 (44.9)	**21.3 (4.2–38.4)**	**3.63**	5.67 (4.46–6.88)	< 0.001*	4.59 (2.42–8.71), < 0.001*				
Stage 3, Resective surgery	20	10 (50.0)	26.0 (1.5–50.5)		4.02 (2.64–5.40)	0.358					
Stage 3, Regenerative surgery	58	25 (43.1)	22.7 (3.5–41.9)		6.00 (4.61–7.40)						
Resective surgery	102	32 (31.4)	57.0 (45.4–68.6)		7.60 (6.79–8.42)	0.444					
Regenerative surgery	125	39 (31.2)	44.2 (39.3–59.1)		7.73 (6.73–8.72)						
Overall	227	71 (31.3)	**47.8 (36.8–58.8)**	**8.96**	7.93 (7.21–8.65)						

* Median survival time for stage 1 could not be calculated because the cumulative survival rate remained higher than 50% until the end of the study.

Note: The category with hazard ratio (HR) = 1.0 was considered the reference category to which other categories were compared; Significant p-values are shown with *. P-value (log-rank test) for comparison between stages was significant based on Bonferroni Correction Method ( stage 1 vs. stage 2, p = 0.012; stage 1 vs. stage 3, p < 0.001; stage 2 vs. stage 3, p = 0.008; < 0.016 = 0.05/3). Cox hazards regression analysis based on surgical technique was not conducted because the p-values from the log-rank test were not statistically significant and did not fulfill the criteria. CI = confidence interval.

Roccuzzo et al. analyzed the 10-year survival rate after peri-implant surgery based on implant type and reported a survival of 55% of TPS implants and 80% of SLA implants. Jemt et al. reported the 3-year, 5-year, and 10-year survival rates of implants treated with peri-implantitis surgery as 94.1%, 85.5%, and 71.6%, respectively [17, 25]. In the present study, the survival rate of surgical treatment was analyzed after classifying it into stage 1, 2, or 3 based on the degree of peri-implant bone loss. The final cumulative survival rate in the present study was 47.8%; stage 1 was 70.7%, stage 2 was 48.9%, and stage 3 was 21.3%. These rates are relatively low compared with other studies. In addition, approximately 50% of all implants were removed at an average of 8.96 years, and 50% of all implants were removed at 8.18 years in stage 2 and at 3.63 years in stage 3 [23, 26]. The low survival rates in the present study may be due to estimating errors in the selection of surgical indications and contraindications as well as the surgeon’s choice of peri-implantitis treatment. However, due to the retrospective study design, the main cause was likely failure to perform strict regular maintenance as time progressed after surgery and the inability to strictly apply the treatment standards.

In the present study, a PD of ≥ 4 mm was used to diagnose peri-implantitis in all stages[8, 27, 28]. In implants, the measured probing values based on the probing force significantly differed compared with natural teeth, and there were many cases where it was difficult to measure the PD depending on the structure of the prosthesis [8]. Although a probing force of 0.25 N is recommended for accurate PD measurement, only a PD ≥ 4 mm was determined as the basic diagnostic condition for peri-implantitis in the present study, as precise application of probing force in actual clinical practice is challenging. In addition, when measuring the exact amount of peri-implant bone loss with a periapical radiograph, numerous errors can occur depending on x-ray angulation, and limitation exists in measuring buccolingual bone loss [8, 20]. Even if the same amount of bone loss occurs, the effect on peri-implantitis can significantly vary depending on the length and form of the implant fixture. Therefore, in this study, only bone loss of ≥ 2 mm was considered a basic diagnostic criterion for peri-implantitis, and stages were classified based only on the ratio of bone loss to fixture length.

Peri-implantitis surgical treatment is classified into resective and regenerative surgery; however, controversy remains among clinicians due to the unpredictable long-term clinical outcome. In several studies, a short-term follow-up was conducted, and regenerative surgery with bone graft was reportedly more advantageous than resective surgery [29–31]. However, in a meta-analysis on long-term clinical outcome, no difference in survival or prognosis of implants was reported between regenerative and resective surgery [32]. In the present study, no statistically significant difference in the clinical outcome of the two treatment methods was observed. In addition, evidence that a specific treatment is more suitable in any severity level of peri-implantitis was not confirmed. Because various bone graft materials and barrier membranes were used in regenerative surgery, the influence on clinical outcome may have been significant. However, in numerous studies, the type of bone graft material used in peri-implantitis regenerative surgery reportedly had no significant effect on clinical outcomes [33–35]. In the present study, implants surgically treated at least twice were excluded from the study because survival analysis based on type of surgical method is not appropriate when different types of surgical treatment are performed for a single implant, and because it is difficult to compare the difference between cases undergoing different numbers of surgical treatment.

In this study, instead of evaluating treatment success through objective indicators such as bone fill or PD loss, only the survival rate was evaluated. Criteria for evaluating success after peri-implantitis treatment are very diverse. Even if the success criteria are met after treatment, the results can be sustained only if maintenance care is regularly and adequately performed [15, 19, 21, 36–38]. In this study, intentionally exposing contaminated and osseointegration-lost implant surfaces and reducing the peri-implant probing depth created an environment where patients could easily perform oral hygiene management. During dental visits, mechanical cleansing of the implant thread area was carried out more clearly using peri-implant curettage and air-powder abrasion (Air-Flow Master®; Shinhung, Korea). However, in actual clinical practice, patients often do not visit for regular maintenance appointments after the discomfort disappears. Consequently, peri-implantitis tends to recur and continue to worsen due to poor oral hygiene care. A comparative clinical study including patients that do and do not follow prescribed maintenance care is needed. However, such studies cannot be performed due to ethical reasons [17, 19, 39]. Since this was a retrospective study, the maintenance care after surgical treatment was not standardized. In addition, there are many cases in which patients use implants without discomfort even if bone loss progresses significantly and would not be included in the success criteria. Therefore, the focus was on survival analysis using the Kaplan-Meier method, taking into consideration factors such as irregular visits or no visits due to the absence of discomfort [40–42].

As this study was conducted retrospectively based on information such as patient files and radiographs, the type of bone dehiscence/configuration, which affects the prognosis of peri-implantitis, could not be accurately investigated [43, 44]. The lack of standardized criteria for performing implantoplasty, usage of barrier membranes, and incomplete records of oral hygiene status evaluation during visits are also shortcomings from the retrospecitve nature. The difficulty of direct comparison with other studies due to differences in definition and classification of peri-implantitis can also be seen as a limitation. In addition, when multiple implants were affected by peri-implantitis in the same patient, possibility that the patient factor affected the prognosis after surgical treatment existed. However, the study result, showing no significant difference in implant survival pattern based on Kaplan-Meier analysis when and when not considering the patient factor should be noted [42]. This study is valuable because it presents criteria for clinicians to classify peri-implantitis severity using general radiographic data and estimate the prognosis of implants after peri-implantitis surgical treatment.

## Conclusions

The survival period and prognosis of implants after surgical treatment for peri-implantitis were clearly distinguished based on the degree of bone loss relative to the length of the implant fixture before surgical treatment. In addition, difference was not found in the prognosis of implants between resective surgery and regenerative surgery, or in the prognosis based on surgical method in any peri-implantitis stage. Thus, regardless of the surgical method used, the bone loss ratio compared with the implant fixture length can be used as a diagnostic tool to determine the prognosis of implant survival after treatment.

## Data Availability

The datasets used and analyzed for the current study are available from the corresponding author on reasonable request.
